# The Role of Interferon Gamma Gene Polymorphism (+874A/T, +2109A/G, and -183G/T) in Response to Treatment Among Hepatitis C Infected Patients in Fars Province, Southern Iran

**DOI:** 10.5812/hepatmon.14476

**Published:** 2014-01-23

**Authors:** Jamal Sarvari, Hossin Norozian, Mohamad Reza Fattahi, Neda Pirbonyeh, Afagh Moattari

**Affiliations:** 1 Department of Bacteriology and Virology, School of Medicine, Shiraz University of Medical Sciences, Shiraz, IR Iran; 2 Gastroenterohepatology Research Center, Shiraz University of Medical Sciences, Shiraz, IR Iran

**Keywords:** Hepatic C, Interferon-gamma, Polymorphism, Genetic

## Abstract

**Background:**

Hepatitis C virus (HCV) infection as a worldwide health problem is associated with cirrhosis and hepatocellular carcinoma. With current treatment regimen, pegylated interferon (PEG-IFN) plus ribavirin, sustain virological response (SVR) is achieved in only 50% of infected individuals. In HCV infection, an inappropriate ratio of cytokines may affect the benefit of antiviral therapy. Given the polymorphisms in regulatory regions of cytokines genes may influence cytokines production.

**Objectives:**

We aimed to investigate both the frequency of genotypes and alleles of interferon gamma (IFN-γ) gene at +874A/T, +2109A/G, and -183G/T loci in HCV-infected patients and their associations with response to therapy.

**Patients and Methods:**

A total of 158 patients were included and treated with PEG-IFN plus ribavirin. The presence of HCV infection in patients was confirmed by reverse transcription polymerase chain reaction, and genomic DNA was extracted from peripheral leukocytes using salting out method. IFN-γ gene polymorphisms were identified by polymerase chain reaction using sequence specific primers and restriction fragment length polymorphism analysis on genomic DNA.

**Results:**

Of 158 patients, 110 (69.5%) subjects achieved SVR and 48 (30.5%) subjects did not respond to therapy. The frequency of AA genotype (P = 0.001; OR: 11.2; CI: 2.26-63.21) and A allele (P = 0.01; OR: 3.23; CI: 1.23 8.56) of IFN-γ gene at +2109 locus were significantly different between the responder and non-responder subjects infected with genotype 1. Regardless of HCV genotype, the frequency of AG genotype was also higher in responder group than those who did not respond to therapy (P = 0.041; OR: 05.05; CI: 1.05-33.25)). In case of IFN-γ gene at +874 locus, there was no difference in genotypes and alleles frequencies between the responder and non-responder subjects infected with HCV genotypes 1 and 3. Haplotype analysis showed no association between haplotypes and response to therapy. All participants had G/T genotype at -183 locus.

**Conclusions:**

Our findings indicate that heterogeneity at +2109 locus of IFN-γ gene but not at +874 locus could interfere with successful therapy in patients infected with HCV genotype 1.

## 1. Background

Hepatitis C virus (HCV) is a major cause of chronic liver disease all-over the world (1). More than 130 million people are chronically infected with HCV who might progress to cirrhosis and hepatocellular carcinoma (2). According to recently published data, the prevalence of HCV is less than 1% in general population (3). With the current treatment regimen, pegylated interferon (PEG-IFN) plus ribavirin, sustain virological response (SVR) is achieved in only 50% of infected individuals (4). A number of factors including age, alcohol consumption, viral genotype, duration of infection, and viral load have been assigned variable significances in determining the response to antiviral treatment (5, 6). However, the emerging field of immunogenetic has confirmed the significant role of heredity in host immune response to infections (7, 8). Considering the importance of immune biomarkers, the beneficial role of cytokines in response to viral infections is well established (9). As a potent inducer of antiviral response, interferon gamma (IFN-γ) a secretary protein mainly produced by T and natural killer cells, has been reported to be involved in the control of HCV replication. Its gene is located on chromosome 12q24.1 and consists of four exons and three introns, which encodes a short polypeptide of 166 amino acids (10). IFN-γ level may interfere and affect the outcome of antiviral therapy in patients with HCV. Accordingly, it has been reported that low level of intrahepatic mRNA of IFN-γ was correlated with response to therapy in chronic HCV infected patients (11). Moreover, another study (12) showed that IFN-γ modulates interferon alpha (IFN-α) therapy by interfering with IFN-α signaling pathways. Also a prior study it was reported that ribavirin may reduce IFN-γ expression and increase the rate of SVR in this way (13). Polymorphisms in cytokines genes have been implicated in several autoimmune and chronic inflammatory conditions (14, 15). There are several polymorphisms located in control and coding region of IFN-γ gene including -G183T, +C764G, +A874T, +A2109G, +G3810A and +G5644A loci (16, 17). In this study, we selected three functional polymorphism sites of the IFN-γ gene that had been previously reported. One polymorphism was located at 874 bp downstream from the translation start site, which coincides with the nuclear factor-κB (NF-κB) binding area (18). The other two were located at 2109 bp downstream and 183 bp upstream from the translation start site which were reported to be involved in transcriptional regulation of IFN- γ gene (19, 20).

## 2. Objectives

The associations between aforementioned polymorphisms and the outcome of several diseases such as tuberculosis, hepatitis B, and brucellosis have been shown in different studies (21-23). To the best of our knowledge, there is no data available from Iran, reporting the effects of these polymorphisms on the outcome of antiviral therapy in patients infected with HCV. Accordingly, in this study we wished to determine the genotypes and alleles frequency of IFN-γ at + 874A/T, +2109A/G and, -183G/T loci among infected patients with HCV, in Fars Province, southern Iran, and to investigate whether they influence the treatment outcome.

## 3. Patients and Methods

### 3.1. Patients

During the study from September 2010 to May 2012, 158 subjects (137 males, 21 females; the mean age of 40.47 years with a range of 19-75 years) were recruited consecutively from Gastroenterohepatology Research Center at Nemazee Hospital in Shiraz. The diagnosis of HCV infection was documented based on biochemical and molecular assays, including the detection of persistent elevation of serum aminotransferase level in the presence of HCV antibody using enzyme immunoassay, and the detection of HCV RNA by reverse transcriptase polymerase chain reaction (RT-PCR). HCV genotypes were analyzed by restriction fragment length polymorphism (RFLP) or genotypes specific primer sets.

All patients received at least 24 weeks of treatment with PEG-IFN alfa-2a, 180 μg weekly, combined with 1000-1200 mg of ribavirin per day. Patients who had detectable HCV RNA at this time received combined therapy for an additional 24 weeks (a total of 48 weeks). Patients who had undetectable HCV RNA 6 months after therapy were included in responder group, and those who had persistent viremia 6 months after the end of therapy were considered as non-responder group. 

Considering the 158 studied patients, 110 subjects were responder and 48 as non-responder. The study was approved by the Ethics Committee of Shiraz University of Medical Sciences, and written informed consent was obtained from each participant before sampling.

### 3.2. Genomic RNA Extraction and Analysis of Cytokine Polymorphisms 

A blood sample of 5 mL was collected from each participant in ethylenediaminetetraacetic acid (EDTA) anticoagulant tubes. DNA was extracted from peripheral blood leukocytes using the salting out procedure. Cytokine gene polymorphisms were studied by polymerase chain reaction (PCR) using a thermal cycler (5530 Master Cycler, Eppendorf, Germany). Polymorphism of IFN-γ gene (at position +874) was identified using allele specific nucleotide polymerase chain reaction (ASO-PCR) as described by Pravica et al. with some modifications (16). Polymorphisms at positions +2109 and -183 of IFN-γ gene were determined with PCR-RLFP based method using the Arthrobacter citreus I and Arthrobacter luteus I restriction enzymes, respectively (20, 21) using the primers shown at Table 1. PCR was performed in a total volume of 25μL, containing 1X reaction buffer (CinnaGen, Iran), 200 μM (each) deoxyribonucleotide triphosphates solution (dNTPs) (CinnaGen, Iran), 1U Taq DNA polymerase ( CinnaGen, Iran), 0.5μM each specific primers and 0.5 and 1.5 mM MgCl2 ( CinnaGen, Iran) for ASO-PCR and PCR-RFLP, respectively. To determine whether PCR amplification was successful, the β-actin-specific primers (F-5' ACA CAA CTG TGT TCA CTA GC-3' and R-5' CAACTT CAT CCA CGT TCA CC-3') were used as an internal control in ASO-PCR. The amplified products were run on 2% agarose gel in a buffer containing 0.5 μg/mL ethidium bromide.

**Table 1.** Primer Sequences and Methods Used for Detection of Cytokine Gene Polymorphisms

Locus	Primers	Method
**IFN +874**[Fn fn6624]	Common primer: 5'-TCA ACA AAG CTG ATA CTC CA-3'	ASO -PCR
	T allele primer: 5'-TTC TTA CAA CAC AAA ATC AAA TCT-3'	
	A allele primer: 5'-TTC TTA CAA CAC AAA ATC AAA TCA-3'	
**IFN +2109**	F5'-AAT CGC TGA AGT ATG TAA T-3'	AciI based RFLP
	R 5'-GCA TTG TAG AGT TTT GCA G-3'	
**IFN -183**	F5'- AAT GTG CTT TGT GAA TGA-3'	AluI based RFLP
	S5'- CTC CTC TGC CTG CTG CTA-3'	

Abbreviations: AciI, Arthrobacter citreus; AluI, Arthrobacter luteus; ASO-PCR, allele-specific oligonucleotide polymerase chain reaction; RFLP, restriction fragment length polymorphism.

### 3.3. Statistical Method

The statistical analyses were performed using EPI-Info 2000 and SPSS version 15 software. Arlequin software package was used for haplotype analysis and also for determining the consistency of the genotypes frequencies with Hardy–Weinberg equilibrium. The data was analyzed by χ^2^ test and P values less than 0.05 were considered as statistically significant. 

## 4. Results

A total of 158 patients with HCV infection were enrolled. The mean age of cases in responder and non-responder groups was 39.18 and 43.14, respectively. The difference between groups was not statistically significant (P = 0.06). No significant difference was found between the groups regarding gender (male to female ratio: 99/11 and 38/10, respectively, P = 0.06). 

### 4.1. IFN-γ Gene Profile in Responder and Non-Responder Groups

The polymorphisms of IFN-γ at +874, +2109, and -183 loci were determined in 128, 134, and 142 patients, respectively. The distribution of cytokine genotypes and alleles of +874 and +2109 loci in all subjects is shown in Table 2. Regardless of HCV genotype, the frequency of AG genotype was also higher in responder group than those who did not respond to therapy (P = 0.041; OR: 05.05; CI: 1.05-33.25). Interestingly, in the case of -183 locus, all studied subjects showed G/G genotype, and no polymorphism was seen at -183G/A locus in our population study. 

**Table 2.** The Frequency of IFN-γ Genotypes/Alleles Between Responder and Non-Responder Groups

IFN-γ Genotype/Allele	Responder, No. (%)	Non-Responder, No. (%)	P value
**+874 AA**	21 (22.8%)	5 (13.5%)	0.51
TT	29 (31.5%)	12 (33.6%)	
TA	42 (45.6%)	19 (52.8%)	
Total	92 (100%)	36 (100%)	
**2109AA**	66 (72.5%)	35 (81.46%)	0.041
GG	7 (7.7%)	6 (13.9%)	
AG	18 (19.8%)	2 (4.1%)	
Total	91 (100%)	43 (100%)	
**+874A**	84 (45%)	29 (40.2%)	0.52
T	43 (59.8%)	100 (55%)	
Total	184 (100%)	72 (100%)	
**+2109A **	150 (80.3%)	72 (80.4%)	0.92
G	32 (19.7%)	14 (19.6%)	
Total	182 (100%)	86 (100%)	

### 4.2. HCV Genotype

The genotype of HCV was determined in 140 of 158 subjects. Sixty-one (43%) subjects were found to be infected with genotype 1, while 71 patients were infected with genotype 3 (47%). Only 4 subjects were infected with genotype 2 (5.12%). The frequency of genotypes 1, 2, and 3 in responder group were 40% (n = 39), 3% (n = 3), and 57% (n = 55), respectively. In non-responder group 22 subjects (50.1%) were infected with genotype 1, whereas 4 and 20 patients were infected with genotype 2 (9.1%) and genotype 3 (46.5%), respectively. Although the difference was not statistically significant, the frequency of genotype 1 in non-responder subjects was higher than those who responded to therapy, and genotype 3 was more prevalent in responder patients than non-responder subjects.

### 4.3. IFN-γ Gene Profile in Responder and Non-Responder Groups with Genotype 1 Infection

For this purpose, the distribution of IFN-γ genotypes and alleles of +874 and +2109 loci were analyzed and compared in both responder and non-responder patients with genotype 1 infection. As indicated in Table 3, among infected patients with HCV genotype 1 no association was found between alleles and genotypes frequency of IFN-γ at +874 locus and response to therapy. However, regarding the +2109 locus, we found an association between the frequency of IFN-γ and response to therapy as shown in Table 3. Interestingly, the frequency of AA genotype (P = 0.001; OR: 11.2; CI: 2.26-63.21) (67% versus 15.8%) and A allele (P = 0.01; OR: 3.23; CI: 1.23 8.56) (77.7% versus 53.7%) was significantly higher in responder groups compared to those who did not respond to therapy. 

**Table 3.** IFN-γ Genotypes/Alleles Frequency Between Responder and Non-Responder Subjects With Genotype 1 Infection, P value Less Than 0.05 Were Considered Statistically Significant

IFN-γ Genotype/Allele	Responder, No. (%)	Non-responder, No. (%)	P value
**+874 AA**	6 (18.7)	1 (6.6)	
TT	7 (21.9)	4 (26.4)	
TA	19 (59.3)	10 (66.6)	
Total	32 (100)	15 (100)	0.5
**+2109AA**	21 (67.7)	3 (15.8)	
GG	3 (9.6 )	2 (10.5)	
AG	7(22.5)	14 (73.6)	
Total	31 (100)	19 (100)	0.0002
**+874A**	31 (46.8)	12 (40)	
T	33 (53.2)	18 (60)	
Total	64 (100)	30 (100)	0.58
**+2109A**	49 (77.7)	21 (53.7)	
G	13 (22.3)	18 (46.3)	
Total	63 (100)	39 (100)	0.01

### 4.4. IFN-γ Gene Profile in Responder and Non-Responder Groups With Genotype 3 Infection

Considering the frequency of genotypes and alleles of IFN-γ at +874 and +2109 loci among infected patients with HCV genotype 3, no statistical differences were observed between responder and non-responder groups as indicated in Table 4. 

**Table 4.** IFN-γ Genotypes/Allele Frequency Between Responder and Non-Responder Subjects With Genotype 3 Infection

IFN-γ Genotype/Allele	Responder, No. (%)	Non-responder, No. (%)	P value
**+874 AA**	11 (34.3)	3 (23)	
TT	4 (53.1)	5 (38.5)	
TA	17 (12.5)	5 (38.5)	
Total	32 (100)	13 (100)	0.14
**+2109AA**	37 (72.5)	15 (83.3)	
GG	3 (5.8)	0 (0)	
AG	11 (21.5)	3 (16.7)	0.53
Total	91 (100)	43 (100)	
**+874A**	11 (42.3)	39 (60.1)	
T	15 (57.7)	25 (38.9)	
Total	64 (100)	27(100)	0.16
**+2109A**	88 (83.7)	30 (84.4)	
G	17 (16.3)	6 (15.6)	
Total	105 (100)	36 (100)	0.84

### 4.5. Demographic and Laboratory Characteristics of Patients With HCV Genotype 1 Infection

Regarding the significant association of IFN-γ polymorphism at +2109A/G with response to therapy in patients with genotype 1 infection, we compared demographic (age and sex) and laboratory (ALT and ST) characteristics of these subjects with response to therapy (Table 5). 

**Table 5.** Comparison of Demographic and Laboratory Characteristics Between Responder and Non-Responder Patients With HCV Genotype 1 Infection

	Responder, No (%)	Non Responder, No (%)	P value
**Age, y**			0.11
≤ 40	22 (56.4)	7 (31.8)	
> 40	17 (43.6)	15 (68.2)	
**Sex**			0.26
Male	35 (89.9)	17 (77.2)	
Female	4 (10.1)	5 (12.8)	
**ALT , IU/L** ^**a**^ **, IU/L**			0.69
≤ 35	14 (39.6)	10 (40.6)	
> 35	25 (60.4)	13 (59.7)	
**AST , IU/L** ^**a**^ **, IU/L**			0.21
≤ 25	12 (31.7)	3 (13.1)	
> 25	26 (58.3)	19 (86.9)	

^a^ Abbreviations: ALT, alanine aminotransferase; AST, aspartate aminotransferase.

### 4.6. Haplotype Analysis

It has been reported that polymorphisms of IFN-γ at +874 and +2109 loci were under linkage disequilibrium. Haplotypes were calculated by Arlequin 3.1 software package (24). A total of four haplotypes (AA, TA, AG, and TG) were constructed, and the frequency of different haplotypes in responder and non-responder groups were respectively as follows: AA: 47.7% versus 68.1%; TA: 37.3% versus 31.9%; AG: 1.4% versus 0%; TG: 13.4% versus 0%. There were no significant associations between different haplotypes and response to therapy.

## 5. Discussion

Several host and viral factors including viral genotype, viral load, age, gender, degree of liver fibrosis, alcohol consumption, and genetic background are shown to be involved in the outcome of response to therapy in patients with HCV infection (8, 22). It seems that cytokines such as IFN-γ may play a role as host genetic factor in disease outcome and response to therapy. It has been reported that polymorphisms in cytokines genes including IFN-γ might have been implicated in several autoimmune and chronic inflammatory conditions such as HCV infection (13, 25).

In this study we showed that the frequency of AA genotype and A allele of IFN-γ +2109A/G polymorphism in responder patients with HCV genotype 1 infection are higher than those who did not respond to therapy. Our results also showed that in patients with genotype 1 infection the frequency of AG genotype in non-responder group is higher than those who responded to therapy (73.6% versus 22.5%). On the other hand, regardless of HCV genotype, we found an association between AG genotype of IFN-γ gene polymorphism at +2109A/G locus with response to therapy. Interestingly, the frequency of AG genotype was higher in responder cases compared to non-responders (19.8% versus 4.6%). However, considering the patients with genotype 3 infection, we could not find such association between genotypes and alleles frequency of this polymorphisms and response to therapy. To the best of our knowledge, there was no data available regarding this polymorphism and response to therapy in patients with HCV infection. Regarding other diseases, a prior study(17) reported that A allele at 2109 locus of IFN-γ gene was less frequent in patients with hepatic schistosomiasis-associated periportal fibrosis than control group, while other studies showed no association between this polymorphism and microscopic-positive tuberculosis (21), and also another study reported that pulmonary tuberculosis was not associated with IFN-γ gene polymorphisms at +2109 locus.

A previous study reported that high level of intrahepatic mRNA of IFN-γ was correlated with no response to therapy in patients with chronic HCV infection (11). Moreover, another study (12) showed that IFN-γ would modulate IFN-α therapy through interfering with IFN-α signaling pathways. Furthermore, one other study (26) reported that ribavirin plus IFN-α might reduce IFN-γ expression that might influence the outcome of anti-HCV therapy. Recently, it has been reported that the serum level of IFN-γ in patients with HCV infection would be higher than healthy people among Iranian individuals (27). Although it has been reported that the polymorphism located at 2109bp locus might be involved in transcriptional regulation of IFN- γ gene (19, 20), it is not yet clear how A to G transition at this polymorphism might influence IFN-γ gene transcription, and as a result, we could not come to a conclusion about the effect of this polymorphism on response to therapy regarding the up/down regulation of IFN-γ gene transcription. 

Another single nucleotide polymorphism (SNP) in IFN-γ gene is located at +874A/T locus. Several studies have been performed on this polymorphism, and it has been associated with hepatitis B virus (HBV) and HCV infections (22, 28), but not with human papilloma virus infection (29) and colorectal cancer (30). Although, some studies in Iran have reported the association of this SNP with pulmonary tuberculosis (31), brucellosis (23), breast cancer (32), asthma (33), and liver transplantation rejection (34), few other studies could not find such association between this polymorphism and HBV infection (35) and recurrent pregnancy loss (36).

In this study we showed that the frequency of alleles and genotypes of IFN-γ at +874 locus were not statistically different in non-responder group compared to responder group among patients with genotype 1 or 3 infection. Our findings are in agreement with the results of a prior study that reported no association of this polymorphism with response to therapy among Pakistani patients with HCV genotype 3 infection (37).

When we compared the frequency of alleles and genotypes at +874A/T locus of IFN-γ gene regardless of HCV genotype, we could not find a statistically significant difference between responder and non-responder patients which is in agreement with the results of another study (38) that reported no association between IFN-γ +874A/T polymorphism and the outcome of antiviral therapy among Tai population with HCV infection. A previously conducted study showed that patients with the T allele high producer genotype +874 IFN-γ, had a significantly higher rate of liver cirrhosis than patients with homozygote A allele (28). It has also been suggested that the presence of high producer alleles (T) might be a genetic risk factor for the development of IFN-α-induced depression in patients with HCV infection treated with IFN-α (39). In another study it has been shown that +764G allele, which is related to high transcriptional level of IFN-γ gene, was more prevalent in responder patients compared to non-responder subjects. Sample sizes, population admixture, differences in ethnic backgrounds, differences in treatment protocols, and different criteria for selection of responder and non-responder groups may all contribute to these conflicting results and discrepancies. The data presented here led us to speculate that in Fars Province of Iran, polymorphism at +874 IFN-γ might not have an impact on achieved SVR in patients with HCV genotypes 1 and 3 infection. 

Analysis of haplotypes based on the linkage disequilibrium between SNPs is a useful tool for identification of predisposing genes of complex diseases. In the present study, haplotypes analysis was performed for each patient to clarify the effect of different haplotypes on SVR (24). It has been reported that the risk of HBV infection in subjects carrying the haplotype AG, which contained the A allele at position +874 and the G allele at position +2109, was 8.14-fold higher compared to those without the haplotype AG (22). In this study we could not find statistically significant differences between haplotypes and response to anti-HCV therapy.

A prior study (19) reported a polymorphism at -183 locus of IFN-γ gene which might influence the AP-1 binding domain and promoter activity, which would influence the transcription and production of IFN-γ. It has also been reported that this polymorphism is associated with chronic HBV infection in Chinese population (40). Interestingly, all of our subjects had GT genotype at -183 of IFN-γ gene, and no polymorphisms were seen in this study group. Although more investigations are needed to be performed, particularly on large study groups; our data supports the rarity of these polymorphisms in our area, Fars Province in Southern Iran.

Consistent with other studies (6, 41), our findings showed that genotype 3 followed by genotypes 1 and 2 were dominant in responder subjects, and genotypes 1 and 3 were the most frequent genotypes in non-responder patients.

It has also been reported that demographic factors such as gender and age could influence the outcome of therapy in patients with HCV infection (5, 42). Although in this study no association was found between gender and response to therapy, the frequency of females who did not respond to therapy was higher than responders (10% versus 20%). Although the mean age of responder subjects was lower than non-responder group, the difference was not statistically significant.

In conclusion, our study showed that in patients with genotype 1 infection, the frequency of AA genotype and A allele were significantly higher in responder subjects compared to non-responder subjects. It would be tempting to speculate that IFN-γ (+2109) AA genotype, as a genetic factor, might influence the response to antiviral therapy among Iranian patients with HCV genotype 1 infection. However, IFN-γ gene polymorphism at +874 and, even perhaps, -183 loci does not seem to have any effect on the outcome of therapy in patients with HCV infection in general population, in Fars Province, Southern Iran.
